# Health in Prison: Does Penitentiary Medicine in Italy Still Exist?

**DOI:** 10.3390/healthcare9111511

**Published:** 2021-11-05

**Authors:** Matteo Bolcato, Vito Fiore, Filomena Casella, Sergio Babudieri, Luciano Lucania, Giulio Di Mizio

**Affiliations:** 1 Legal Medicine, University of Padua, Via G. Falloppio 50, 35121 Padua, Italy; 2 Department of Medical, Surgical and Experimental Sciences, Infectious and Tropical Diseases Clinic, University of Sassari, 07100 Sassari, Italy; vitofiore30010516@gmail.com (V.F.); babuder@uniss.it (S.B.); 3 Department of Legal Medicine, Sant’Anna e San Sebastiano Hospital, 81100 Caserta, Italy; mena.casella84@gmail.com; 4 Italian Society of Penitentiary Medicine (SIMSPE), 30165 Rome, Italy; luciano.lucania57@gmail.com; 5 Unit of Penitentiary Medicine of Reggio Calabria, 81100 Reggio Calabria, Italy; 6 Forensic Medicine and Criminology, Department of Law, Magna Graecia University of Catanzaro, 88100 Catanzaro, Italy; giulio.dimizio@unicz.it

**Keywords:** health, prison, prison medicine, therapeutic alliance, clinical risk management, informed consent, legal medicine

## Abstract

Despite the detailed legislative developments that have occurred within the context of prison medicine in Italy, problems of a management nature continue to affect prisoner health and management, which in turn impact the prison system’s ability to offer prisoners a real opportunity for rehabilitation. Certain behavioral aspects reported in prisons may alter and negatively impact the normal doctor-patient relationship, including elements that hinder the therapeutic alliance and impede proper clinical risk prevention and management. However, practical steps may be taken in connection with the analysis of flows and healthcare services that may enable prison administrations to bring about a true, modern restructure of the prison system.

## 1. Introduction

Health is universally considered a human right that must be defended and guaranteed [1,2,3]. In Italy, it has been protected by the Constitution since 1948, so much so that Art. 32 of the Constitution states: “The Republic protects health as a fundamental right of the individual and collective interest and guarantees free medical care to patients”. Numerous legislative provisions have been enacted to uphold this principle with a view to providing solidarity and universality in supporting the Constitution and maintaining the *Servizio Sanitario Nazionale* (hereinafter SSN) or National Health Service. More recently, Law No. 24/2017 [4,5] was enacted and integrated the safety of care [6,7] as a fundamental element of the right to health.

These rights do not cease to apply to those who have been incarcerated in a prison facility. On the contrary, the objective of Italian law is to facilitate rehabilitation during the period of incarceration. In order to achieve that objective, human rights must be respected, including healthcare and treatment for prisoners through the prison medical system. This service is necessary not just in terms of public health, for example to contain diseases, infectious or otherwise; rather, it serves as an integral part of rehabilitation, re-educating prisoners in legality, society, and respect for the rule of law.

The organization of this system, however, is a complex undertaking, both as regards treatment on an individual level with the associated issues in terms of the need for restrictions and therefore providing said treatment in a non-hospital setting, and due to the inevitable psychological and behavioral implications that this specific method of care and treatment entails.

## 2. The Law

The first legislative document regarding prison institutions to be issued in Italy dates back to 1891 and is entitled: “Regulations for Prison Facilities and Governmental Reformers”. The document was founded on the positivist school of criminology, which defined treatment as “differentiated, scientific, and personalized”, the linchpin of prison politics, and focused its attention on the human and social reality of the prisoner. The document made a distinction between “prison facilities” and “reformatory facilities”, thus beginning to create a primitive differentiation in prisoner treatment based on their age and legal status at the time. Therefore, the idea began to prevail that the focus of sentence execution should be on prisoner rehabilitation. Regulation No. 787 of 18 June 1931, entitled “Regulations for Prevention and Penal Institutions” [8], is included in a Royal Decree and is the first document (Articles 304, 305, 306, p66, third part) to identify doctors as part of prison personnel and define their responsibilities.

No new regulations were issued on the topic of prison healthcare until 1970 with the promulgation of Law No. 740/1970, which began to regulate, though only partially, the intramural health service; it also defined the role of “appointed doctor”, i.e., the doctor who renders services in prison but is not part of the Prison Administration. During that time, healthcare services were “mutualistic” in nature, and hospitals were “autonomous entities”, financed by “mutual funds”.

Thereafter came a breakthrough in the history of the Italian healthcare regulatory framework with the institution of the *Servizio Sanitario Nazionale Italiano* (*SSN*) (Italian National Health Service) by means of Law No. 833/1978. This fundamental provision placed the focus on citizens and their healthcare needs and identified the necessary stages of prevention, diagnosis, treatment, and rehabilitation. This legislation did not cover prison healthcare, which continued to be reliant on the Ministry of Justice wherein doctors were employed and specifically appointed but were not part of the SSN, which took care of free people. That prison healthcare system, still tied to an antiquated model of reacting to the need for control rather than the need for care and treatment, began to feel the effects of SSN intervention in the prison world.

In 1990, Presidential Decree No. 309/90 then entrusted SSN doctors with the management of drug addicts, and Law No. 296/1993 defined the internal management protocols for prisoners affected by HIV (rooms for prisoners in SSN hospitals equipped with departments of infectious diseases). The first real turning point came in 1998: Law No. 419/1998 set down the transfer of prison healthcare management to the SSN. It took 10 long years for the transfer to take effect, finally approved by the Prime Minister’s Decree dated 1 April 2008. This decree finalized the transfer to the SSN and stipulated the transition method, which was conclusive and represented a true reform, since management of the entire prison health service, which cared for between 50 and 60,000 prisoners, was transferred from the prison administration to the National Health Service.

## 3. Prison Health Service and Prisoner Health Management: Art. 11 of the Italian Prison Law as a Functional Tool

Recently, Legislative Decree No. 123, dated 2 October 2018, introduced new instruments to amend Italian Prison Law, specifically Art. 11, which remains the regulator of prison medical care [9]. The article sets out that “the Italian SSN operates within prison institutions, as set forth in the direction on the reorganization of prison medicine, and provides that each prison institution be equipped with healthcare services that meet prisoners’ needs. Each Local Health Authority with a prison institution located within its jurisdiction adopts the charter of healthcare services referred to in Legislative Decree No. 230, dated 22 June 1999, which must be made available to prisoners by means of appropriate advertising methods”. As a result, the following are responsible for prisoner health, each within their own remit: prison doctors, the Department of Prison Administration, the Courts, and Public Prosecutors.

Furthermore, Article 11 sets out that “in the event that treatments or medical tests that cannot be performed by the prison healthcare services are required, inmates are to be transferred to external healthcare facilities for diagnosis or treatment on the authorization of the competent court”. In addition, jurisdiction over rulings regarding prisoner healthcare varies depending on the stage of proceedings, as indicated in Table 1 below.

On entry into prison, the prisoner undergoes a general medical exam, following which the prison doctor provides him or her with comprehensive information regarding his or her health status. The doctor immediately records in the medical file all information regarding signs or indicators of violence or maltreatment and forwards such information to the prison governor and jurisdictional authority. Prisoners also have the right to receive comprehensive information regarding their health status throughout the period of incarceration and at the time of their release. Throughout the period of detention, healthcare services are provided periodically on the basis of the prisoners’ health needs and in line with the principles of proactivity, comprehensive prevention of health risks, consistency of services and performance, involvement of social and healthcare services, and ensured continuity of care. The doctor’s role is to ensure daily medical exams for sick prisoners and for those who request them, in line with the clinical need.

This approach means that healthcare services are available to whomsoever should require them in the event of illness in the same way that free people visit their general practitioner or undergo diagnosis for a condition. Such organizational developments throughout the period of incarceration enable the health needs of the prison population to be determined and handled with an ever-increasing level of care, from basic prison healthcare and Intensive Care to “first generation” hospital departments (where the prison model prevails over the hospital model) and finally to “protected medicine” (an autonomous department within the hospital used to receive prisoners who require longer admission in an external treatment center), where a specialist nucleus of Prison Police, in conjunction with the Prison Administration, operates 24/7 to ensure the completion of all healthcare activities. This “protected medicine” provides hospitalized prisoners with all the specialist services available in the hospital by collaborating with other hospital departments, ensuring a high level of care safety for prison patients.

In addition, the prison health service must ensure that prisoners, on remand or after being sentenced, who are currently undergoing therapy for the rectification of sex (pursuant to Law No. 164, dated 14 April 1982), may continue said therapy and receive the necessary psychological support.

A further complex issue addressed by the law concerns granting prisoners permission to be examined by a healthcare professional of their choice at their own expense. Using the same methods, authorization can be given for medical, surgical, and therapeutic treatments to be carried out by doctors and technicians of their choice at the expense of the person concerned in the infirmary or clinical and surgical departments within the institution, subject to agreement with the Local Health Authority and compliance with said authority’s organizational directions. This option, which is appropriate considering the guarantees that the prison population should be offered, is available if in line with the specific prisoner’s healthcare needs and treatment recommendations supported by scientific evidence.

## 4. Prison Administration Roles and Tools

Health services in Italian prisons fall under the management of both the Ministry of Justice and the Ministry of Health, and are then devolved to the 20 regional authorities. The regulatory body is identified in the Guidelines provided by the Joint Conference Agreement between Regions and Autonomous Provinces dated 22 January 2015 on the following theme: Guidelines on Methods for Delivering Health Services in Adult Prison Institutions; Implementation of Regional and National Health Networks [10]. The health service that handles prison healthcare, therefore, is governed by agreements devised in Joint Conference, which are then enacted by the regions and Local Health Departments by means of specific legislation; Local Health Authorities, in turn, proceed with the practical implementation of such legislation.

There appears to be a fundamental difference between the management and organizational methods utilized by the SSN and the Prison Administration. The SSN is not governed by a hierarchical management system, even though the Ministry of Health has indicated Essential Assistance Levels (Livelli Essenziali di Assistenza) or LEA, which comprise the services that the SSN is required to provide to all citizens throughout the national territory. The regional authorities then have the task of developing organizational and management processes to deliver those services; therefore, strategic planning is in large part devolved to the local authorities with wide autonomy.

The Prison Administration, however, operates under a different organizational scheme. The head of this vertical management structure has authority over the entire prison system, which is managed according to a strict hierarchy. The Directorates General and Regional Superintendents exercise jurisdiction over correction facilities. It is a state system that is rigid and has precisely defined roles, tasks, and responsibilities.

These two very different worlds must coexist: the health service—required to follow Guidelines and Best Practices and comply with SSN agreements, overcome challenges related to clinical risks and safety of care in line with Law No. 24, 8 March 2017, on the safety of care, in addition to bringing the internal system in line with the latest requirements regarding informed consent and legal institutions with a view to ensuring respect for patient self-determination [11] as set forth in Law No. 219, 22 December 2017 [12]—meets and on occasion clashes with the prison system—a rigid, complex, and hierarchical organizational structure entrusted with the care of those deprived of their personal freedoms. This rigidity often becomes problematic for the healthcare system.

The healthcare and welfare services, combined, are highly structured and complex, and, when fully functioning, enable prisoners to be transferred from one prison to the next to receive better care. The objective of this network is to manage detainees in the most appropriate way for their conditions. The prison healthcare service is, *and even more so following the pandemic*, a true medical department and is equipped to provide a plethora of services.

## 5. Therapeutic Alliance and Consent to Treatment

Article 3 of the Charter of Fundamental Rights of the European Union states: “Right to the integrity of the person. Everyone has the right to respect for his or her physical and mental integrity. In the field of medicine and biology, the following must be respected in particular: (a) the free and informed consent of the person concerned, according to the procedures laid down by law; (b) the prohibition of eugenic practices, in particular those aiming at the selection of persons; (c) the prohibition on making the human body and its parts as such a source of financial gain; (d) the prohibition of the reproductive cloning of human beings.” Article 11 regarding freedom of expression and information states: “Everyone has the right to freedom of expression. This right shall include freedom to hold opinions and to receive and impart information and ideas without interference by public authority and regardless of frontiers. 2. The freedom and pluralism of the media shall be respected”.

Unconditional consent given after information has been provided is therefore essential to the performance of any acts on the physical and psychological integrity of the person.

Article 5 of the Convention on Human Rights and Biomedicine (Oviedo Convention), enacted on 4 April 1997, states: “1. An intervention in the health field may only be carried out after the person concerned has given free and informed consent on it. 2. This person shall beforehand be given appropriate information as to the purpose and nature of the intervention as well as on its consequences and risks. 3. The person concerned may freely withdraw consent at any time.” Article 10, Private life and right to information, states: “2. Everyone is entitled to know any information collected about his or her health. However, the wishes of individuals not to be so informed shall be observed”.

Italian Law No. 219/2017, dated 22 December 2017, on “Provisions for informed consent and advance treatment directives” provides an extraordinary contribution to the issue of information and consent [13]. Article 1 states: “This law, in compliance with the principles set out in Articles 2, 13, and 32 of the Constitution and Articles 1, 2, and 3 of the Charter of Fundamental Rights of the European Union, protects the right to life, health, dignity, and self-determination of the person and establishes that no health treatment can be started or continued without the free and informed consent of the person concerned, except in cases expressly provided for by law. 2. The relationship of care and trust between patient and doctor is promoted and valued, which is based on informed consent in which the patient’s decision-making autonomy and the doctor’s expertise, professional autonomy, and responsibility coincide. Healthcare professionals who make up the healthcare team contribute to the care relationship based on their respective skills. 3. Everyone has the right to know their health condition and to be fully informed, updated and understand the diagnosis, prognosis, benefits and risks of the diagnostic tests and health treatments indicated, as well as the possible alternatives and consequences of any refusal of medical treatment and diagnostic assessments or withdrawal from such”.

In the following articles, the law sets out the tools at the patient’s disposal to make treatment choices in advance by drawing up an advance medical directive or a shared care plan. The law states that the patient has the right to be informed on several aspects: health condition, diagnosis, prognosis, benefits, and risks of the diagnostic tests and health treatments indicated, as well as the possible alternatives and consequences of any refusal of recommended treatment [14].

The law explicitly states that a patient must receive information in a complete, up-to-date, and comprehensible manner. Therefore, the doctor–patient relationship model should include a suitable method of communication for the patient’s condition and ability in addition to being centered on the patient’s needs and situation, without compromising its complete and up-to-date nature. Methods of communication that are incomplete, misleading, and aimed at obtaining consent to the procedure without real patient involvement must be avoided.

Respect for these principles in the prison environment has proven to be a significant challenge, especially due to the peculiar nature of the relationship created between patient/prisoner and prison general practitioner/healthcare director. The prison healthcare director assumes the role of general practitioner from the moment of the prisoner’s arrival in prison. The relationship may initially appear to mirror the normal characteristics of a free person–general practitioner relationship, but, in reality, it is subject to the self-serving use of medical, pharmacological, and welfare resources available to prisoners. The prison context inevitably obliges prisoners to adhere to internal rules and even procedures for which prison benefits may legitimately be requested [15].

Prisoners soon realize that a sickness may enable them to take advantage of a less burdensome prison regime and even house arrest on provision of evidence of the inadequacy of the internal prison healthcare system to treat their particular sickness. It is possible, then, for prisoners to exacerbate any conditions they may suffer from [16] or even simulate pathological symptoms [17] in order to elude their current confinement [18,19]. The work of a prison doctor is therefore somewhat complex: obviously, he or she must always prioritize prisoner health but can never rule out, in the event of incoherent clinical elements, that the observational data he or she is presented with may be the result of incongruous choices on the part of the prisoner. Only a high level of efficiency and adequacy on the part of the prison healthcare services will enable the appropriate management of the complex reality of the prisoner–doctor relationship. Consequently, the internal healthcare system may find itself under pressure from guarantors of prisoner rights and their lawyers, who—appropriately—are concerned for the health of detainees, without understanding the effect the incongruous behavior of their clients has on the actual situation. Connected to this is the matter of the information that is provided to prisoners regarding their sickness and to the related consent that prisoners may or may not give to needed treatment.

## 6. Process for Admitting Incarcerated Patients: Roles and Methods

Admitting a patient entails devising a healthcare plan and arranging, on the basis of clinical criteria and the patient’s specific needs [20,21,22], for the appropriate appointments. The Prison Medical Director is responsible for planning personalized treatment programs and scheduling all necessary healthcare services, in accordance with internal processes, within the appropriate timeframe—both for basic and specialist health services. This takes place initially on reception into prison and should continue throughout the organization and reorganization of activities related to patient diagnosis, treatment, and periodic checks. In many areas in Italy, this is performed by means of electronic processes, since healthcare departments are subdivisions of the health service in each region.

The admission process is fundamental both for young and old prisoners, both for those who enter prison in moderate health, who should undergo preventive checks, and for chronically ill patients. It should never be forgotten that medicine is first a relationship between two people—doctor and patient—and then a healthcare system, an organization, and a business. Prison healthcare has become a matter of importance not only for professionals within the justice system but also in the public opinion in that it represents a paradox, an antimony, since the two concepts can seem conflicting. Rehabilitation of the person, sociability, and relationships cannot be accomplished through a sentence for a crime, which we impose as a state, unless accompanied with care and respect for the offender’s health. By means of the sentence, prison, as a place of both health and suffering, must facilitate reformation [23]. Consequently, health is to be viewed as a building block of said reformation, and thus the health of each person must be considered on reception: from those with no issues to those with medium to extensive issues. The prison’s capacity to provide healthcare should be equal to the healthcare needs of each prisoner.

In light of the experience acquired since the implementation of the 2008 Decree, in order to provide those deprived of their liberty with optimal healthcare, protected medicine units, which are currently only present in certain regions, should be installed across the entire national territory. The function of these units, or hospital departments, should not be limited to simply supervising people with health problems, but should be designed to provide prisoners with the complex and integrated services that form the foundation of healthcare today. Similarly, the state should set forth the option to utilize appropriate prison facilities to handle prisoners with particularly complex health issues safely, especially the disabled.

## 7. Clinical Risk in Prisons: Reality or Myth?

Objective clinical risks do exist, not as a result of an illness a prisoner is affected by in itself but of the structural and residential context [24,25]. Clinical risk procedures can only be applied to the internal health service, not to the entire prison complex, due to the fact that prisons are not hospitals; they are containers that house a subdivision of the SSN. The prison system is affected by the same healthcare management issues as the national system with the added challenges of performance and patient safety indicators that can be falsified or unreliable, precisely because not all diagnostic-therapeutic regimens proceed as planned but often meet with obstacles and attempts to manipulate the system for personal gain. This risk is closely related to the “breakdown” of the therapeutic alliance in that if the prison doctor is seen as an antagonist, or at the very least a hindrance to the patient’s healthcare needs, the relationship may cease to function, leading to a lack of correspondence between the data recorded in the medical file and reality, incongruence between drug consumption and actual needs [26,27,28], and a superfluity of diagnostic tests. However, these obstacles should not be viewed as an indicator that the implementation of clinical risk management programs in prison healthcare departments represents an unachievable chimera and therefore should not be set as an objective; on the contrary, despite the fact that detailed processes and strict supervision would be required, clinical risk management is fundamental considering the higher risks present in this context.

## 8. Conclusions

This article has examined only a fraction of the issues and problems that affect the complex relationship between prisoners and the prison health service, but it has highlighted the complex reality of a system that is showing signs of extraordinary need and potential. The prison system, though unique as an institution detached from the external world, in essence is faced with the same logistical challenges as any other type of organization with its internal departments, human resources (in this case represented by prison staff), its mission (i.e., to facilitate prisoner rehabilitation), problems of communication and conflict between the various roles, and the continual need for mediation.

In Italy, over 60,000 people are currently confined to a prison environment and assisted by the prison health service, which has been given new lifeblood by the SSN and its new vision of healthcare management, risk prevention, and ability to ensure essential assistance levels throughout the country. The real challenge, therefore, is to optimize the organization of healthcare treatment programs for the prison population in line with the SSN’s ability. The schemes used by the national health service can be adapted and integrated with other strategies in order to achieve that objective. For example, the flow of services can be tracked within the prison environment, be they of an instrumental, physical, or pharmacological nature, and can therefore be analyzed with a view to ascertaining the prison population’s welfare needs and determining the organization’s response or remedial action in the event said flow corresponds to appropriate or self-serving ends. It also appears necessary to further study sex differences regarding health in prison, given the profound imbalance between the numbers of male and female inmates [29].

In all cases, even the most difficult, the solution lies in system analysis [30] through data; only then can proper system management be achieved [31].

Innovation in this regard is not only to be considered as a response to the healthcare needs of a prison population [32] but shows, from a legal perspective, that an adequate and thorough health service, even in the prison environment, promotes personal and social development mechanisms for prisoner rehabilitation and the prevention of recidivism. As a result, it also diminishes the cost of prisons on society.

In conclusion, not only for medical but also for ethical and legal reasons, an overhaul of the organization of prison healthcare in Italy is needed.

## Figures and Tables

**Table 1:** Jurisdictional authority in the protection of prisoner health throughout the various stages of proceedings.

Stages of proceedings	Jurisdictional authority
Prior to Prosecution	Examining Magistrate
Collegiate Court	Judgment Issued by the President
Summary Judgment	Public Prosecutor
For the Convicted	Supervisory Magistrate

## Data Availability

Not applicable.
